# Island vs. Mainland: Genetic Divergence of *Calotes versicolor* (Daudin, 1802) (Squamata: Agamidae) in Thailand

**DOI:** 10.3390/ani15203028

**Published:** 2025-10-19

**Authors:** Bhuvadol Gomontean, Warayutt Pilap, Chavanut Jaroenchaiwattanachote, Panida Laotongsan, Pichit Pliankham, Jatupon Saijuntha, Wittaya Tawong, Chairat Tantrawatpan, Weerachai Saijuntha

**Affiliations:** 1 Department of Biology, Faculty of Science, Mahasarakham University, Kantharawichai District, Maha Sarakham 44150, Thailand; bhuvadol.g@msu.ac.th; 2 Walai Rukhavej Botanical Research Institute, Mahasarakham University, Maha Sarakham 44150, Thailand; warayutt@msu.ac.th (W.P.); panida.la@msu.ac.th (P.L.); 3 Center of Excellence in Biodiversity Research, Mahasarakham University, Maha Sarakham 44150, Thailand; chavanut.j@msu.ac.th; 4 Administration Program in Public Administration, Faculty of Political Science and Public Administration, Rajabhat Maha Sarakham University, Maha Sarakham 44000, Thailand; pichit@rmu.ac.th; 5 Faculty of Engineering, Mahasarakham University, Maha Sarakham 44150, Thailand; jatupons2534@gmail.com; 6 Department of Agricultural Sciences, Faculty of Agriculture Natural Resources and Environment, Naresuan University, Phitsanulok 65000, Thailand; wittayat@nu.ac.th; 7 Center of Excellence in Biodiversity, Center of Excellence in Research for Agricultural Biotechnology, Naresuan University, Phitsanulok 65000, Thailand; 8 Division of Cell Biology, Department of Preclinical Sciences, Faculty of Medicine, and Center of Excellence in Stem Cell Research and Innovation, Thammasat University, Rangsit Campus, Pathum Thani 12120, Thailand; 9 Biomedical Science Research Unit, Faculty of Medicine, Mahasarakham University, Maha Sarakham 44000, Thailand

**Keywords:** mitochondrial *CO1*, population structure, haplotype diversity, phylogeography, Agamidae, genetic variation

## Abstract

**Simple Summary:**

Islands provide unique opportunities to study how geographic isolation influences genetic diversity and evolutionary processes. The Oriental Garden lizard (*Calotes versicolor*) is a widespread reptile found across mainland and island habitats in Thailand. By analyzing mitochondrial *CO1* sequences from populations in the Andaman Sea and Gulf of Thailand regions, we assessed their genetic diversity and population structure. The results revealed a mixture of shared and unique haplotypes, with some island and mainland populations being genetically similar, while others showed clear divergence. These patterns suggest that both historical connections and current geographic barriers have shaped the genetic landscape of *C. versicolor*. Our findings contribute to the understanding of reptile biogeography in Southeast Asia and provide valuable information for biodiversity conservation.

**Abstract:**

Geographic isolation can shape genetic variation and structure, leading to divergence between island and mainland populations. The Oriental Garden lizard (*Calotes versicolor* Daudin, 1802) is a widespread agamid reptile in Asia, occurring across diverse habitats from continental Southeast Asia to offshore islands. We examined mitochondrial cytochrome c oxidase subunit I (*CO1*) sequence variation in 143 individuals from 23 localities across the Andaman Sea and Gulf of Thailand to assess genetic diversity and structure between insular and mainland populations. Forty-six haplotypes (Cve1–Cve46) were identified, with haplotype diversity (Hd) ranging from 0.500 to 1.000 and nucleotide diversity (π) from 0.0057 to 0.0265. AMOVA revealed low to moderate differentiation between island and mainland groups in the Andaman Sea (*F*_CT_ = 0.075, *p* > 0.05) and negligible differentiation in the Gulf of Thailand (*F*_CT_ = 0.009, *p* > 0.05). Haplotype networks and PCoA showed clustering of most island and mainland populations within regions, with some localized divergence. Divergence-time analysis indicated that lineages split within the last 0.5 million years ago (Ma), coinciding with late Pleistocene climatic oscillations and sea-level changes. Species delimitation analyses supported three major lineages, including a geographically restricted clade confined to Trat Province and Phuket Island. These results suggest that *C. versicolor* populations are structured more by regional geography than strict island–mainland separation, reflecting historical connectivity and contemporary gene flow. The findings contribute to understanding reptile biogeography in Southeast Asia and highlight populations of conservation value.

## 1. Introduction

Investigating genetic variation between mainland and island populations is essential for understanding the evolutionary dynamics, biogeographic history, and conservation needs of reptile species [1]. Islands, under their geographic isolation, often foster unique evolutionary processes such as genetic drift, founder effects, and local adaptation, which can lead to significant genetic divergence from mainland populations [2,3]. In contrast, mainland populations usually maintain higher gene flow and more stable genetic structures. Comparative genetic studies between these populations provide key insights into patterns of diversification, speciation, and adaptation [4,5]. Such studies are especially important in biodiversity hotspots like Southeast Asia, where geological complexity and ecological heterogeneity have shaped the evolutionary histories of many terrestrial vertebrates [6,7,8].

*Calotes versicolor* (Daudin, 1802), the oriental garden lizard, is an ecologically adaptable agamid reptile widely distributed across South and Southeast Asia. It inhabits a range of environments, from forests and agricultural landscapes to urban areas [9,10]. Despite being regarded as a single widespread species, *C. versicolor* exhibits notable morphological, behavioral, and genetic variation across its range, suggesting possible cryptic diversity and regional adaptation [11,12]. Island populations of *C. versicolor*, in particular, may have undergone distinct evolutionary trajectories compared to their mainland counterparts due to prolonged isolation and environmental differences.

Molecular genetic tools, especially mitochondrial DNA markers such as cytochrome c oxidase subunit 1 (*CO1*), 16S ribosomal RNA (16S rRNA), and cytochrome *b* sequences (*Cyt-b*), have proven effective in revealing hidden genetic structure, lineage diversification, and phylogeographic patterns in *C. versicolor* [13,14,15,16]. When coupled with spatial landscape data, these tools can help identify environmental or geographic features that facilitate or impede gene flow. By comparing genetic variation between island and mainland populations of this species within a landscape genetics framework, researchers can uncover how historical biogeography, natural selection, and contemporary landscape features contribute to population divergence and potentially to speciation.

Recent studies have emphasized the extent of cryptic genetic diversity within the *C. versicolor* complex [10,13,15,16]. For example, *Calotes* populations from Hainan Island, China, have been recognized as a distinct species, *Calotes wangi hainanensis*, based on both morphological and mitochondrial DNA evidence [17]. Similarly, a study of *C. versicolor* populations along the Mekong River in Thailand and Lao PDR revealed the existence of at least six genetically distinct mitochondrial lineages (C–H), suggesting that cryptic speciation may also be occurring within continental populations of Southeast Asia. Interestingly, this study found no significant genetic barrier effect of the Mekong River, highlighting the importance of geographic distance over physical barriers in shaping population structure in this species [15].

Comparing island and mainland populations provides a powerful lens to explore how geographic isolation, ecological differences, and limited gene flow drive divergence. Island populations often exhibit reduced genetic diversity and stronger effects of drift or local adaptation, while mainland populations typically retain greater connectivity. Understanding these dynamics helps reveal cryptic diversity and guides conservation priorities. However, no study has yet conducted a systematic comparison of island and adjacent mainland *C. versicolor* populations across the Thai archipelagos. Our study addresses this gap by examining mitochondrial *CO1* sequence variation between insular and mainland populations in the Andaman Sea and Gulf of Thailand, providing the first comprehensive assessment of island–mainland genetic divergence of *C. versicolor* in this region.

## 2. Materials and Methods

### 2.1. Sample Collection

**Figure 1:**
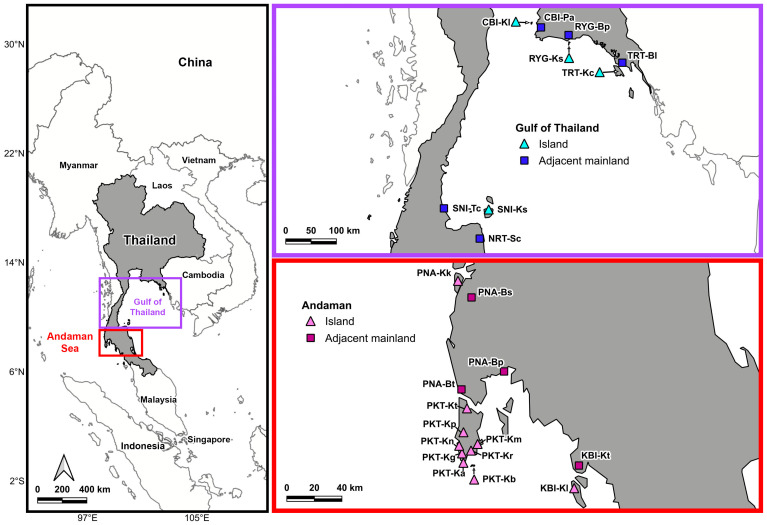
Map showing the 23 collection localities of *Calotes versicolor* across the Andaman Sea and the Gulf of Thailand.

Since all *Calotes* agamid lizards, including *C. versicolor*, are protected animals by law in Thailand, we obtained approval to conduct this research through the Department of National Parks, Wildlife and Plant Conservation of Thailand. A total of 88 samples were collected from 14 localities on islands in the Gulf of Thailand and the Andaman Sea, including 55 samples from 9 localities in adjacent mainland areas (Table 1 and Figure 1). The *C. versicolor* were captured by fishing pole method [18]. Buccal epithelial cells were collected using the buccal swab method described by Koutsokali et al. [19]. Each swab was gently rubbed against the inner cheek and oral epithelium for approximately 6 s to obtain cellular material. All specimens were released at their original collection sites immediately after buccal swabbing. Swabs were immediately placed into tubes containing TE/SDS buffer and stored on ice in the field before transfer to −20 °C in the laboratory for DNA extraction. 

### 2.2. Molecular Analysis

Buccal swabs from each individual lizard were used for DNA extraction using the E.Z.N.A.^®^ Tissue DNA Kit (Omega Bio-tek, Norcross, GA, USA), following the manufacturer’s instructions. Swabs were first thawed, vigorously vortexed to release epithelial cells, and then removed. The adhered material was washed with TE buffer and added back to the original tube before proceeding with DNA extraction. The extracted DNA was then used as a template to amplify the *CO1* gene using primers and PCR conditions described in a previous study [15]. All PCR products, approximately 1500 bp in size, were gel-purified using the E.Z.N.A.^®^ Gel Purification Kit (Omega Bio-tek, Norcross, GA, USA). The purified PCR products were then sent for DNA sequencing at ATGC Co., Ltd. (Pathum Thani, Thailand).

### 2.3. Data Analyses

All 1258-bp *CO1* sequences generated in this study were aligned using the ClustalW program version 2.0 [20], and variable sites among haplotypes were examined using the BioEdit program version 7.2.5 [21]. Molecular diversity indices, haplotype data, and mismatch distribution analysis were calculated using the DnaSp program version 5.0 [22]. Haplotype network was constructed in the Network program version 10.2 (https://www.fluxus-engineering.com/; accessed on 25 July 2025) based on a median-joining network [23] using all sequences generated in this study. Analysis of Molecular Variance (AMOVA) and genetic differentiation (Φ_ST_) analyses were conducted using the Arlequin program version 3.5.2.2 [24]. A spatial analysis of molecular variance (SAMOVA) version 1.0 was applied to cluster the *CO1* sequences into genetically and geographically homogeneous groups [25]. SAMOVA partitions variance into K groups using the AMOVA framework and estimates variation among groups (*F*_CT_), variation among populations within group (*F*_SC_), and variation among individuals within population (*F*_ST_). Analyses were performed for K = 2–8 with 1000 simulated annealing steps from 100 random starting conditions. Principal coordinate analysis (PCoA) was conducted in GenAIEx 6.5 [26] using pairwise Nei’s genetic distance to assess interpopulation affinities. Isolation-by-barrier (IBB) patterns were evaluated in R version 4.5.0 [27] by plotting Φ_ST_ values between populations within the same habitat type (island or mainland) and between different habitat types (island vs. mainland).

### 2.4. Time-Calibrated Phylogenetic Tree

To estimate the phylogeny and divergence times for the 143 *CO1* sequences of *C. versicolor*, we conducted a Bayesian analysis using the program BEAST version 2.7.7 [28]. Using a strict molecular clock with a mean substitution rate of 5.87% per site per million years, estimated by a previous diversification and demography study of *C. versicolor* [13]. The most appropriate evolutionary model was selected in MrModeltest version 2.4 [29], in which GTR+I+G was selected by the Akaike Information Criterion (AIC) method. A coalescent constant population prior was used for the tree. Samples from the posterior were drawn every 1000 steps over a total of 3 × 10^7^ MCMC steps. The analysis was checked for MCMC convergence in Tracer version 1.7.2 [30]. The initial 10% of steps was discarded as burn-in and visualized in Figtree version 1.4.4. [31]

### 2.5. Species Delimitation Analysis

Two phenetic, single-locus species delimitation methods were applied to the *C. versicolor CO1* dataset: Automatic Barcode Gap Discovery (ABGD) [32] and Assemble Species by Automatic Partitioning (ASAP) [33]. Both analyses were conducted using the bioinformatic toolkit iTaxoTools v0.1 [34]. For ABGD, the Kimura (K80) substitution model was employed, with the default maximum and minimum intraspecific distances (Pmax = 0.1, Pmin = 0.001). The barcode gap width was set to 1.5. Since recursive partitions appeared over-split, a non-recursive partition was selected, with a prior maximal distance of *p* = 7.77 × 10^−3^. For ASAP, the partition with the lowest ASAP score and an appropriate threshold distance (dT) was chosen under the Kimura (K80) model, using a default transition/transversion ratio of 2.0 and minimum and maximum threshold distances of 0.05 and 0.5, respectively.

## 3. Results

### 3.1. CO1 Sequence Variation

A total of 143 individuals from 23 populations were analyzed to assess genetic variation based on mitochondrial *CO1* sequences, which have been deposited in GenBank under accession numbers PX308698–PX308840. Across six population groups defined by SAMOVA (Table S1), 161 polymorphic sites and 46 haplotypes were identified (Table 2). Genetic diversity varied among groups. Group A (*n* = 25) contained 18 polymorphic sites and 10 haplotypes, with high haplotype diversity (Hd = 0.797 ± 0.076) but low nucleotide diversity (π = 0.0043 ± 0.0003). Group B (*n* = 5) showed the highest nucleotide diversity (π = 0.0355 ± 0.0072) and complete haplotype diversity (Hd = 1.000 ± 0.126), based on 82 segregating sites and five haplotypes. Group C (*n* = 3) exhibited moderate haplotype diversity (Hd = 0.667 ± 0.314) and low nucleotide diversity (π = 0.0027 ± 0.0013). Group D (*n* = 85), the largest sample, had 71 segregating sites, 27 haplotypes, and high diversity (Hd = 0.932 ± 0.013; π = 0.0076 ± 0.0006). Group E (*n* = 7) contained 76 segregating sites but only three haplotypes, resulting in moderate haplotype diversity (Hd = 0.524 ± 0.209) and relatively high nucleotide diversity (π = 0.0245 ± 0.0092). In contrast, Group F (*n* = 18) was monomorphic, with a single haplotype (Hd = 0.000; π = 0.0000). Overall, haplotype diversity across all groups was very high (Hd = 0.948 ± 0.008), and nucleotide diversity reached 0.0180 ± 0.0014.

### 3.2. Genetic Differentiation

**Figure 2:**
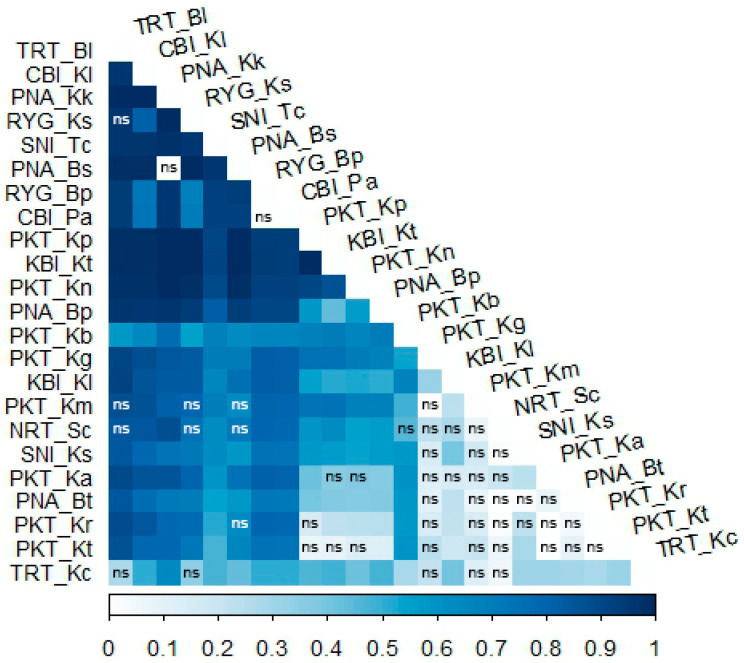
Heat map showing genetic difference represented by Φ_ST_ values based on *CO1* sequences among populations of *Calotes versicolor*. The x-axis represents Φ_ST_ values ranging from 0 to 1, corresponding to variation in color shedding. The y-axis represents the locality codes of *C. versicolor* populations. The abbreviation ‘ns’ denotes no significant difference (*p*-value ≥ 0.05), while other values represent significant genetic differences (*p*-value < 0.05). Locality codes are provided in Table 1.

The pairwise genetic differentiation (Φ_ST_) heat map illustrated the genetic differences among *C. versicolor* populations from various island and mainland localities in Andaman Sea and Gulf of Thailand (Figure 2). Populations from Phuket Province, a biggest island in Thailand, exhibited low genetic divergence and potential gene flow. In contrast, several comparisons between island and mainland populations, revealed weak or non-significant correlations (Φ_ST_ < 0.2, *p* > 0.05), suggesting notable genetic divergence. These findings support a pattern of isolation in insular populations. The presence of numerous non-significant (ns) pairwise correlations, primarily involving island populations, further underscoring their distinct genetic composition. Notably, populations from islands in northern Gulf of Thailand (e.g., TRT-Kc, RYG-Ks, and CBI-Kl) consistently displayed significant genetic differences with the adjacent mainland populations (TRT-Bl, RYG-Bp, and CBI-Pa), indicating limited gene flow between populations.

### 3.3. Genetic Structure

Analysis of molecular variance (AMOVA) was conducted to measure genetic differentiation between island and mainland populations of *C. versicolor* in both the Andaman Sea and Gulf of Thailand regions. In the Andaman Sea region, only a small portion of the total genetic variation was explained by differences between island and mainland groups (*F*_CT_ = 0.07395, *p* > 0.05), indicating minimal genetic separation at this level. Most genetic variation occurred among populations within each group (*F*_SC_ = 0.62462, *p* < 0.001), and there was also substantial variation within individual populations. The overall genetic differentiation was high (*F*_ST_ = 0.59686, *p* < 0.001), reflecting considerable genetic diversity across populations regardless of island or mainland status. By comparison, populations from the Gulf of Thailand showed a somewhat higher degree of genetic differentiation between island and mainland groups (*F*_CT_ = 0.14827, *p* > 0.05), suggesting a moderate effect of geographic grouping here. However, as in the Andaman Sea region, the majority of genetic variation was still found among populations within groups (*F*_SC_ = 0.72971, *p* < 0.001), along with significant variation within populations themselves (*F*_ST_ = 0.68963, *p* < 0.001). These results indicate that while there is some genetic differentiation between island and mainland populations most genetic variation exists at the population level rather than strictly between island and mainland groups.

The Spatial Analysis of Molecular Variance (SAMOVA) based on *CO1* data revealed clear genetic structuring of *C. versicolor* across island and mainland populations. The proportion of variation among groups (*F*_CT_) ranged from 0.58234 at K = 3 to 0.67849 at K = 8 (Table S1, Figure S1). Although the highest *F*_CT_ value at K= 8 suggested over-fragmentation, *F*_CT_ reached a plateau at K = 6 (*F*_CT_ = 0.66885, *p* < 0.001). At this level, variation among populations within groups declined sharply (*F*_SC_ = 0.30753, *p* < 0.001) (Table 3).

**Figure 3:**
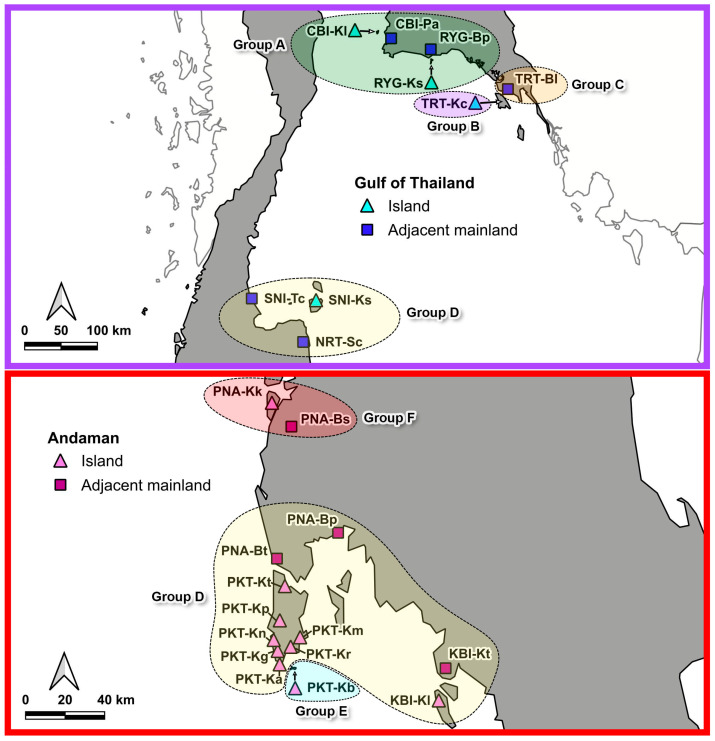
Clustering of genetically and geographically homogeneous populations among 23 populations of *C. versicolor* across Andaman and Gulf of Thailand using the Spatial Analysis of Molecular Variance (SAMOVA) at K = 6.

The SAMOVA analysis identified six genetic clusters corresponding broadly to the Andaman Sea and Gulf of Thailand regions (Figure 3). In the Gulf of Thailand, four clusters were detected: group A (RYG-Ks, RYG-Bp, CBI-Kl, CBI-Pa), group B (TRT-Kc), group C (TRT-Bl), and group D (SNI-Tc, SNI-Ks, NRT-Sc). Group A included both island and mainland populations, while group D showed affinities with some Andaman populations. Groups B and C, each represented by a single population, were genetically distinct from their neighbors. In the Andaman region, three clusters were identified: group D (island and mainland populations from both regions), group E (southern Phuket Island, PKT_Kb), and group F (one Andaman Island and an adjacent mainland population). Group D, the most complex, indicates historical gene flow between regions. Overall, genetic differentiation was strongest between the Andaman Sea and Gulf of Thailand, whereas island and mainland populations within each region generally shared similar genetic backgrounds.

**Figure 4:**
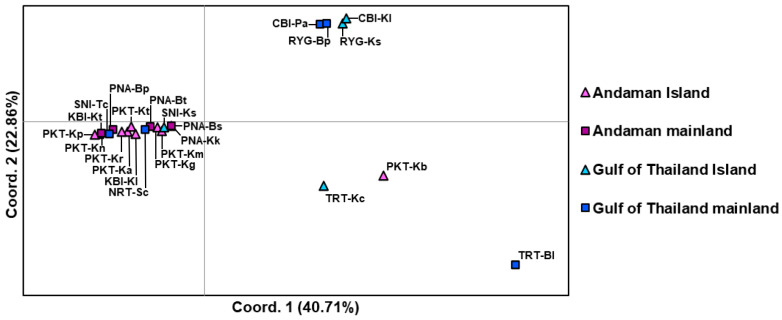
The interpopulation genetic affinity of 23 populations of *Calotes versicolor* based on *CO1* data; two-dimensional scatter diagram based on principal coordinate analysis.

The Principal Coordinate Analysis (PCoA) based on genetic distances revealed some variation among populations of *C. versicolor* from both mainland and island locations (Figure 4). The first two coordinates accounted for 63.57% of the total genetic variation, with 40.71% and 22.86% explained by the first and second axes, respectively. Most island populations in the Andaman Sea and adjacent mainland populations clustered closely together, indicating minimal genetic differences and suggesting moderate genetic similarity between island and mainland groups. A few populations—such as TRT-Bl from the Gulf of Thailand mainland, TRT-Kc from a Gulf of Thailand island, and PKT-Kb from an island in Andaman Sea, were somewhat separated, representing localized divergence rather than a broad pattern. Additionally, some island populations from the Gulf of Thailand grouped closely with certain Andaman mainland populations, suggesting historical connectivity or gene flow. These findings indicate no significant genetic differentiation between mainland and island populations, with most groups exhibiting substantial genetic similarity despite geographic separation.

**Figure 5:**
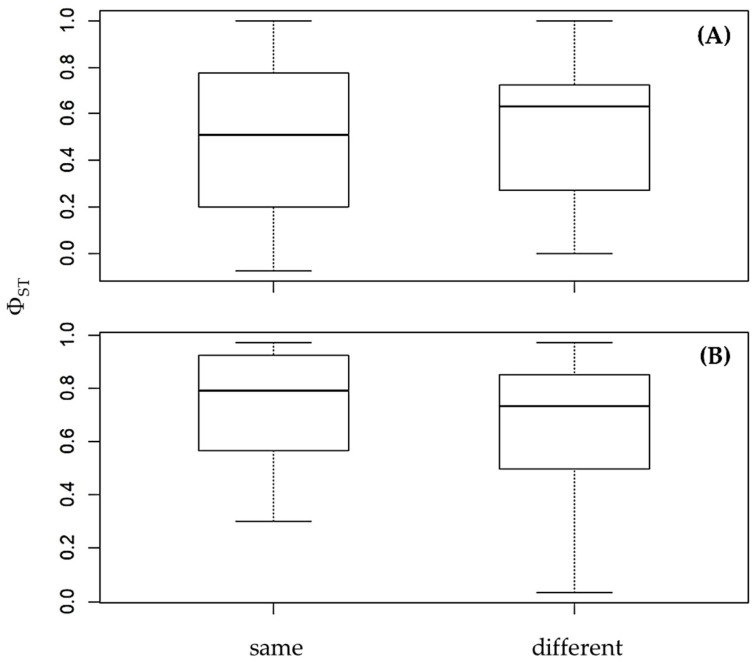
Graph showing isolation-by-barrier patterns between island and adjacent mainland populations of *Calotes versicolor* in the Andaman Sea (**A**) and the Gulf of Thailand (**B**). Pairwise genetic distances (Φ_ST_) are categorized as either “different” (mainland vs. island) or “same” (within mainland or within island), with seawater boundaries recognized as natural barriers to gene flow.

Pairwise genetic distances (Φ_ST_) showed no clear difference between island–mainland (different) and same-landmass (same) comparisons in either region (Figure 5). In the Andaman Sea (Figure 5A), median Φ_ST_ values were similar between the two categories, suggesting that seawater boundaries do not strongly restrict gene flow. Likewise, in the Gulf of Thailand (Figure 5B), both categories exhibited consistently high Φ_ST_ values, indicating that factors other than seawater separation, such as historical isolation or limited dispersal, may be the primary drivers of population genetic structure in this region.

### 3.4. Haplotype Network

**Figure 6:**
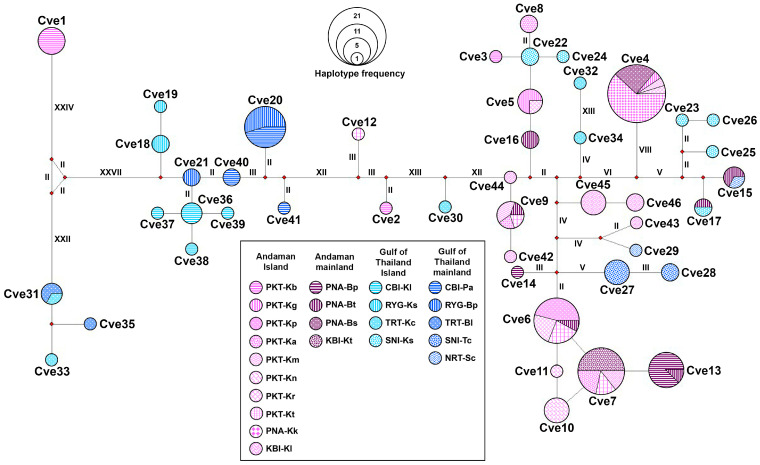
Haplotype network of *Calotes versicolor* generated based on partial *CO1* sequences corresponds to 46 haplotypes (Cve1–Cve46) from 23 different localities across the Andaman Sea and the Gulf of Thailand. Different printed patterns and colors in the haplotype networks represent the various localities examined in this study. The area of the circles represents the proportion of specimen numbers found in each haplotype. The length of each branch is indicated by Roman numerals representing the number of mutational steps (ms), with values greater than one displayed.

The haplotype network based on partial *CO1* sequences revealed clear genetic structuring of *C. versicolor* across islands and adjacent mainland regions of Thailand (Figure 6). A total of 46 haplotypes (Cve1–Cve46) were identified. Several haplotypes (e.g., Cve4, Cve6, and Cve20) were shared between island and mainland populations, indicating recent divergence or gene flow. In contrast, many haplotypes were restricted to specific regions, such as Cve31 and Cve33 in the Gulf of Thailand mainland and Cve1 in the Andaman Islands, suggesting local differentiation. The most frequent haplotypes (Cve4, Cve6, and Cve20) occupied central positions in their respective clusters and were surrounded by numerous low-frequency and singleton haplotypes, forming star-like patterns typical of recent population expansion.

### 3.5. Divergence Time and Species Delimitation

**Figure 7:**
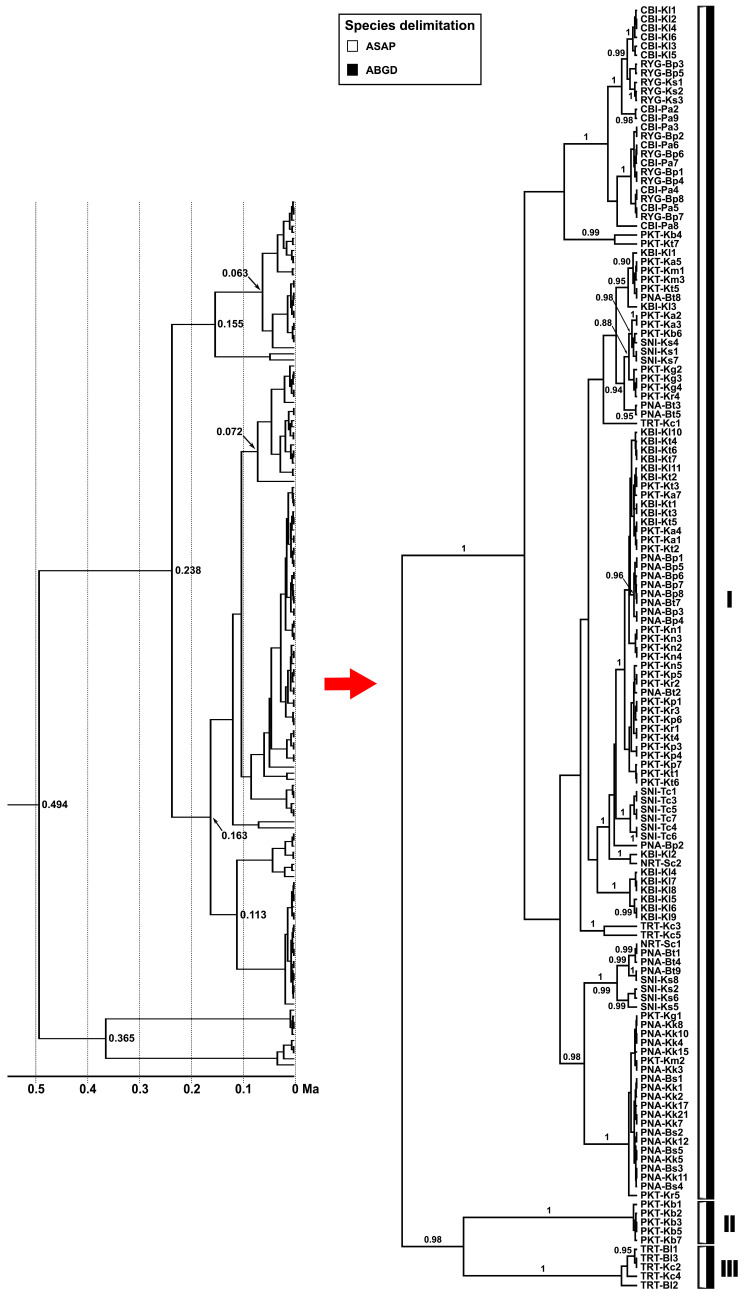
Time-calibrated phylogenetic tree and species delimitation results based on partial *CO1* sequences of *Calotes versicolor* from islands and adjacent mainland regions of Thailand. Three major clades (I–III) were identified by species delimitation analyses. Ma = million years ago.

The time-calibrated phylogeny based on *CO1* sequences revealed that *C. versicolor* populations from islands and adjacent mainland regions of Thailand diverged relatively recently, within the last 0.5 million years (Ma) (Figure 7). The deepest split among the sampled lineages was estimated at ~0.49 Ma, with subsequent diversification events occurring at ~0.36, 0.24, 0.16, and 0.11 Ma. Most nodes were strongly supported, with posterior probabilities ranging from 0.90 to 1.00, indicating high confidence in the branching topology. Species delimitation analyses using ASAP and ABGD consistently identified three major genetic clusters. Clade I included the majority of populations examined, reflecting a wide distribution across both islands and adjacent mainland areas. Clade II was restricted to a subset of populations, including PKT and TRT, whereas Clade III was exclusively represented by individuals from Ko Chang Island and the adjacent mainland in Trat Province (TRT). Both delimitation methods yielded nearly identical species boundaries, supporting the robustness of these groupings.

## 4. Discussion

This study provides the first systematic comparison of mitochondrial *CO1* sequence variation between island and mainland populations of *C. versicolor* in Thailand. We found high haplotype diversity (Hd = 0.500–1.000) and moderate nucleotide diversity (π = 0.0046–0.0245), indicating that populations retain substantial genetic variation, consistent with the wide ecological tolerance and broad distribution of the species [10,13]. Such genetic diversity has also been reported in other broadly distributed agamids [35,36] and is often associated with long-term persistence across varied habitats.

Clustering of some island and mainland populations, particularly in the Gulf of Thailand, suggests historical connectivity facilitated by Pleistocene sea-level fluctuations. During glacial maxima, sea levels dropped by ~120 m, exposing land bridges that enabled dispersal between islands and adjacent mainland regions [37,38]. Divergence-time analyses indicate that lineage diversification occurred within the last 0.5 Ma, coinciding with late Pleistocene climatic oscillations and sea-level changes. The presence of identical haplotypes across both regions is consistent with this scenario, reflecting either retention of ancestral polymorphisms from recent divergence or occasional over-water dispersal that has maintained genetic similarity. Comparable patterns of shallow divergence between islands and nearby mainland populations have been reported in other Southeast Asian reptiles and amphibians, including *Varanus salvator* [39], *Gekko gecko* [40,41], and *Bronchocela cristatella* [42].

In *C. versicolor*, the presence of identical haplotypes across Gulf of Thailand islands and adjacent mainland localities, as well as between the Andaman Sea islands and nearby mainland sites, suggests either ongoing over-water dispersal, possible in species capable of rafting or surviving short saltwater crossings, or recent divergence without sufficient time for mutation accumulation. Such genetic similarity indicates that these populations are either still connected through occasional gene flow or have not been separated long enough for substantial divergence to occur. Although such dispersal events are generally rare, they have been documented in lizards from island archipelagos [43,44]. This pattern aligns with the relatively low and non-significant genetic differentiation observed in our AMOVA, IBB, and PCoA analyses. Similarly, evidence from the Mekong River indicates that it does not function as a significant natural barrier for *C. versicolor* [15], further supporting the species’ ability to maintain genetic connectivity across geographic features that might restrict dispersal in other reptiles.

Unique haplotypes restricted to particular islands suggest long-term isolation and independent evolutionary trajectories. Similar patterns have been documented in *Draco* lizards of Southeast Asia, where island populations often form distinct genetic lineages [45,46]. Over evolutionary timescales, such isolation can potentially promote genetic divergence that may lead to cryptic speciation, as demonstrated by *C. versicolor* populations from Hainan Island, which show distinct genetic divergence from nearby mainland populations [13]. These Hainan populations have subsequently been described as a new species, *Calotes wangi*, with a new subspecies, *C. w. hainanensis* [17]. Therefore, our findings may indicate that *C. versicolor* populations inhabiting isolated islands, particularly in Phuket Island in the Andaman Sea, are on similar evolutionary paths that could ultimately result in speciation. Our findings raise the hypothesis that *C. versicolor* populations inhabiting isolated islands, particularly in Phuket Island in the Andaman Sea, may be on similar evolutionary paths. However, this requires confirmation using multilocus nuclear markers, morphology, and ecological evidence.

From a conservation perspective, populations with high divergence or unique haplotypes (e.g., TRT-Kc in the Gulf of Thailand and PKT-Kb in the Andaman Sea) represent potential evolutionarily significant units (ESUs) [47,48]. Protecting such lineages is crucial to preserving the evolutionary potential of *C. versicolor*. Conservation strategies should prioritize habitat protection, genetic monitoring, and preventing the introduction of non-native reptiles that may hybridize with local forms. Given the broad range of *C. versicolor* and evidence of deep intraspecific genetic structuring in other parts of its range [10,12,13,15], including further taxonomic investigation using multilocus genomic data, may reveal unrecognized lineages in Thailand. Integrating morphological, ecological, and genomic analyses could clarify whether the observed divergence reflects population-level variation or cryptic species boundaries.

## 5. Conclusions

Our analysis of mitochondrial *CO1* sequences in *C. versicolor* populations across islands and adjacent mainland areas in Thailand revealed a mosaic of genetic patterns. Some island and mainland populations remain genetically similar, likely due to historical land connections and possible ongoing dispersal, while others exhibit localized divergence shaped by long-term isolation. Although sample sizes were limited for some populations and only a mitochondrial marker was used, the results underscore the combined influence of historical processes and contemporary barriers on population structure. Conservation of *C. versicolor* should address both widespread interconnected lineages and geographically restricted, genetically distinct populations, which may represent ESUs. Future research integrating nuclear genomic data, morphology, and ecological information will be essential to refine the evolutionary history of this species and to support evidence-based conservation strategies.

## Figures and Tables

**Table 1:** Sampling localities and other related details for *Calotes versicolor* populations collected in Southeast Asia.

Sea	Island						Adjacent Mainland					
	Province	Locality	Code	N	Latitude	Longitude	Province	Locality	Code	N	Latitude	Longitude
**Andaman**	Phuket	Banana beach	PKT-Kb	7	7.744250 N	98.382056 E	Phang-nga	Bang Pat	PNA-Bp	8	8.363444 N	98.577556 E
		Kata beach	PKT-Kg	4	7.817139 N	98.299611 E		Ban Tha Noon	PNA-Bt	8	8.207417 N	98.295056 E
		Patong beach	PKT-Kp	6	7.885417 N	98.277472 E						
		Promthep Cape	PKT-Ka	6	7.761278 N	98.309889 E						
		Muaeng district	PKT-Km	3	7.865389 N	98.398889 E						
		Karon beach	PKT-Kn	5	7.850500 N	98.292861 E						
		Rawai beach	PKT-Kr	5	7.770861 N	98.319750 E						
		Talang district	PKT-Kt	7	8.120278 N	98.333694 E						
	Phang-nga	Ko Kho Khao	PNA-Kk	13	8.959778 N	98.274194 E	Phang-nga	Bang Saphan	PNA-Bs	5	11.313972 N	99.467389 E
	Krabi	Ko Lanta	KBI-Kl	11	7.685361 N	99.109583 E	Krabi	Klong Tom	KBI-Kt	7	7.920472 N	99.251694 E
**Gulf of Thailand**	Chonburi	Ko Lan	CBI-Kl	6	12.914111 N	100.777861 E	Chonburi	Pattaya	CBI-Pa	8	12.840167 N	100.948722 E
	Rayong	Ko Samed	RYG-Ks	3	12.551417 N	101.447389 E	Rayong	Ban Phe	RYG-Bp	8	12.631500 N	101.444694 E
	Trat	Ko Chang	TRT-Kc	5	12.078250 N	102.368194 E	Trat	Ban Laem Kho	TRT-Bl	3	12.171976 N	102.394995 E
	Surat Thani	Ko Samui	SNI-Ks	7	9.568056 N	100.010611 E	Surat Thani	Tha Chana	SNI-Tc	6	9.588056 N	99.207361 E
							Nakhon Sri Thammarat	Sichon	NRT-Sc	2	9.044722 N	99.849361 E
**Total**				**88**						**55**		

**Table 2:** Molecular diversity indices of *Calotes versicolor* from different geographical localities in islands and the adjacent mainland of Thailand based on *CO1* sequence analysis.

Populations	*n*	S	H	Uh	Hd ± SD	π ± SD
Group A	25	18	10	10	0.797 ± 0.076	0.0043 ± 0.0003
Group B	5	82	5	4	1.000 ± 0.126	0.0355 ± 0.0072
Group C	3	5	2	1	0.667 ± 0.314	0.0027 ± 0.0013
Group D	85	71	27	26	0.932 ± 0.013	0.0076 ± 0.0006
Group E	7	76	3	3	0.524 ± 0.209	0.0245 ± 0.0092
Group F	18	0	1	0	0.000 ± 0.000	0.0000 ± 0.0000
Total	143	161	46	36	0.948 ± 0.008	0.0180 ± 0.0014

*n*, sample size; S, segregation site; H, number of haplotypes; Uh, unique haplotype; Hd, haplotype diversity; π, nucleotide diversity; SD, standard deviation.

**Table 3:** Analysis of Molecular Variance (AMOVA) and Spatial Analysis of Molecular Variance (SAMOVA) based on *CO1* sequences of *Calotes versicolor* from different geographical localities, with groups defined by the populations in the island and adjacent mainland in Andaman Sea and Gulf of Thailand.

Source of Variation	d.f.	Ss	Vc	%Va	Fi
**Island vs. Mainland (Andaman Sea)**					
Among groups	1	19.812	0.59221	7.40	*F*_CT_ = 0.07395
Among populations within groups	12	467.403	5.37169	67.08	*F*_SC_ = 0.62462 *
Within populations	81	261.490	3.22827	40.31	*F*_ST_ = 0.59686 *
**Island vs. Mainland (Gulf of Thailand)**					
Among groups	1	25.902	2.04137	14.83	*F*_CT_ = 0.14827
Among populations within groups	7	442.639	11.5359	83.79	*F*_SC_ = 0.72971 *
Within populations	39	166.646	4.27299	31.04	*F*_ST_ = 0.68963 *
**Six groups estimated by SAMOVA**					
Among groups	5	956.288	10.4062	66.88	*F*_CT_ = 0.66885 *
Among populations within groups	17	224.554	1.58446	10.18	*F*_SC_ = 0.30753 *
Within populations	120	428.136	3.56780	22.93	*F*_ST_ = 0.77068 *

d.f., degree of freedom; Ss, Sum of squares; Vc, Variance components; %Va, Percentage of variation; Fi, Fixation index; * *p*-value < 0.001; Six groups (A to F) defined by SAMOVA analysis as shown in Figure 3.

## Data Availability

All data are available upon request.

## Supplementary Materials

The following supporting information can be downloaded at: https://www.mdpi.com/article/10.3390/ani15203028/s1, Table S1: Statistically proposed grouping by Spatial Analysis of Molecular Variance (SAMOVA). Figure S1: Fixation indices obtained by SAMOVA.
