# Supplementary material for: Island vs. Mainland: Genetic Divergence of Calotes versicolor (Daudin, 1802) (Squamata: Agamidae) in Thailand
Source: Animals (Basel). 2025 Oct 19;15(20):3028. doi: 10.3390/ani15203028 (PMC12562272; doi:10.3390/ani15203028)
Supplement: Supplementary file 1 [file animals-15-03028-s001.zip › Figure S1.pdf]

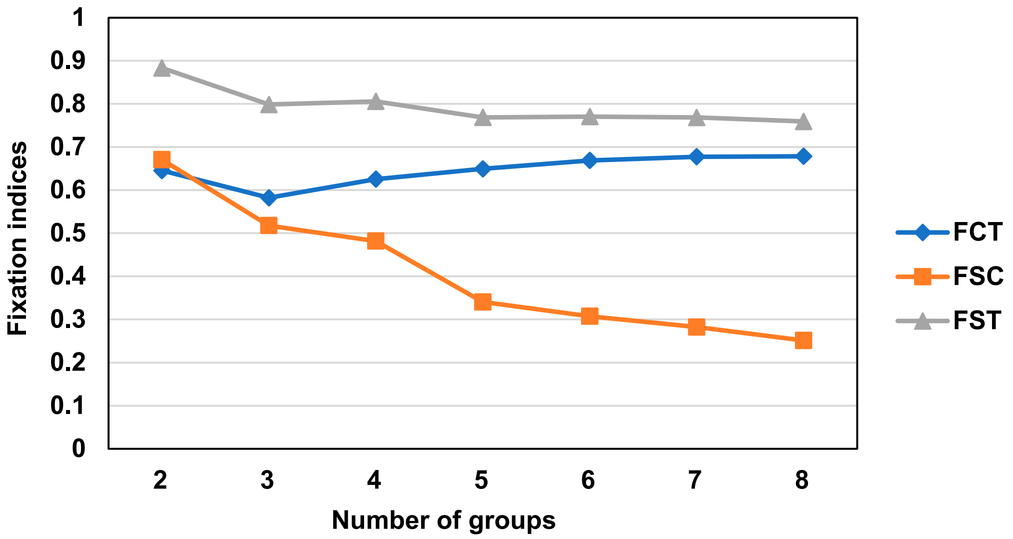

Figure S1. Fixation indices obtained by SAMOVA for the best-clustering option at each pre-defined values of K.
