# Supplementary material for: Island vs. Mainland: Genetic Divergence of Calotes versicolor (Daudin, 1802) (Squamata: Agamidae) in Thailand
Source: Animals (Basel). 2025 Oct 19;15(20):3028. doi: 10.3390/ani15203028 (PMC12562272; doi:10.3390/ani15203028)
Supplement: Supplementary file 1 [file animals-15-03028-s001.zip › Table S1.pdf]

Table S1. Statistically proposed grouping by spatial AMOVA (SAMOVA).

Number of groups	Proposed group composition	F_{CT}	F_{SC}	F_{ST}
2	(RYG_Ks, RYG_Bp, CBI_Kl, CBI_Pa, PKT_Kb, PKT_Kg, PKT_Kp, PKT_Ka, PKT_Km, PKT_Kn, PKT_Kr, PKT_Kt, PNA_Bp, SNI_Ks, SNI_Tc, NRT_Sc, TRT_Kc, KBI_Kl, KBI_Kt, PNA_Kk, PNA_Bs, PNA_Bt) (TRT_Bl)	0.64550*	0.67066**	0.88325**
3	(RYG_Ks, RYG_Bp, CBI_Kl, CBI_Pa) (PKT_Kg, PKT_Kp, PKT_Ka, PKT_Km, PKT_Kn, PKT_Kr, PKT_Kt, PNA_Bp, SNI_Ks, SNI_Tc, NRT_Sc, TRT_Kc, KBI_Kl, KBI_Kt, PNA_Kk, PNA_Bs, PNA_Bt) (PKT_Kb, TRT_Bl)	0.58234**	0.51801**	0.79869**
4	(RYG_Ks, RYG_Bp, CBI_Kl, CBI_Pa) (PKT_Kb) (PKT_Kg, PKT_Kp, PKT_Ka, PKT_Km, PKT_Kn, PKT_Kr, PKT_Kt, PNA_Bp, SNI_Ks, SNI_Tc, NRT_Sc, TRT_Kc, KBI_Kl, KBI_Kt, PNA_Kk, PNA_Bs, PNA_Bt) (TRT_Bl)	0.62555**	0.48189**	0.80599**
5	(RYG_Ks, RYG_Bp, CBI_Kl, CBI_Pa) (PKT_Kb) (PKT_Kg, PKT_Kp, PKT_Ka, PKT_Km, PKT_Kn, PKT_Kr, PKT_Kt, PNA_Bp, SNI_Ks, SNI_Tc, NRT_Sc, KBI_Kl, KBI_Kt, PNA_Bt) (PNA_Kk, PNA_Bs) (TRT_Kc, TRT_Bl)	0.64943**	0.34047**	0.76878**
6	(RYG_Ks, RYG_Bp, CBI_Kl, CBI_Pa) (PKT_Kb) (PKT_Kg, PKT_Kp, PKT_Ka, PKT_Km, PKT_Kn, PKT_Kr, PKT_Kt, PNA_Bp, SNI_Ks, SNI_Tc, NRT_Sc, KBI_Kl, KBI_Kt, PNA_Bt) (PNA_Kk, PNA_Bs) (TRT_Kc) (TRT_Bl)	0.66885**	0.30753**	0.77068**
7	(RYG_Ks, CBI_Kl) (RYG_Bp, CBI_Pa) (PKT_Kb) (PKT_Kg, PKT_Kp, PKT_Ka, PKT_Km, PKT_Kn, PKT_Kr, PKT_Kt, PNA_Bp, SNI_Ks, SNI_Tc, NRT_Sc, KBI_Kl, KBI_Kt, PNA_Bt) (PNA_Kk, PNA_Bs) (TRT_Kc) (TRT_Bl)	0.67752**	0.28244**	0.76860**
8	(RYG_Ks, RYG_Bp, CBI_Pa) (CBI_Kl) (PKT_Kb) (PKT_Kg, PKT_Kp, PKT_Ka, PKT_Km, PKT_Kn, PKT_Kr, PKT_Kt, PNA_Bp, SNI_Ks, NRT_Sc, KBI_Kl, KBI_Kt, PNA_Bt) (PNA_Kk, PNA_Bs) (SNI_Tc) (TRT_Kc) (TRT_Bl)	0.67849**	0.25115**	0.75923**

Significant was tested by 1000 permutations (* *p*-value < 0.05, ** *p*-value < 0.001). Group are represented in parentheses.
